# Comparative study of [^18^F]AlF-NOTA-FAPI-RGD and [^18^F]FDG/[^18^F]AlF-NOTA-FAPI-04 PET/CT in renal cell carcinoma

**DOI:** 10.7150/thno.113070

**Published:** 2025-04-21

**Authors:** Haiyan Gao, Zhiwei Ma, Ziyang Zhu, Zhichuan Yang, Bo Chen, Xiaoming Wu, Vivianne Jakobsson, Yujiao Deng, Hao Wang, Wei Zhang, Jingjing Zhang

**Affiliations:** 1 Department of Nuclear Medicine, Sichuan Provincial People's Hospital, School of Medicine, University of Electronic Science and Technology of China, Chengdu 610072, China.; 2 Department of Urology, Sichuan Provincial People's Hospital, University of Electronic Science and Technology of China, Chengdu, 610072, China.; 3 Department of Emergency Surgery, The Affiliated Chengdu 363 Hospital of Southwest Medical University, Chengdu 610041, China.; 4 College of Nuclear Science and Technology, Harbin Engineering University, Harbin 150001, China.; 5 Department of Diagnostic Radiology, Yong Loo Lin School of Medicine, National University of Singapore, Singapore, 119074, Singapore.; 6 Theranostics Center of Excellence, Yong Loo Lin School of Medicine, National University of Singapore, 11 Biopolis Way, Helios, Singapore 138667, Singapore.; 7 Clinical Imaging Research Centre, Centre for Translational Medicine, Yong Loo Lin School of Medicine, National University of Singapore, Singapore 117599, Singapore.; 8 Nanomedicine Translational Research Program, Yong Loo Lin School of Medicine, National University of Singapore, Singapore, 117597, Singapore.

**Keywords:** [18F]AlF-radiolabeling, Renal cell carcinoma, Fibroblast activation protein, Integrin αvβ3, FAPI-RGD

## Abstract

Renal cell carcinoma (RCC) is a significant global health concern, and the early diagnosis and accurate staging of clear cell renal cell carcinoma (ccRCC) remain major challenges. [^18^F]FDG PET/CT is not ideal for diagnosing ccRCC due to the low glucose metabolism potential of cancer cells. Both fibroblast activation protein (FAP) and the angiogenic integrin α_v_β_3_ receptor are closely linked to the pathogenesis and progression of ccRCC. The aim of this study is to evaluate a novel radiopharmaceutical [^18^F]AlF-NOTA-FAPI-RGD (denoted as [^18^F]AlF-LNC1007), a dual-targeting heterodimer tracer targeting both FAP and integrin α_v_β_3_, and to compare the diagnostic value of [^18^F]AlF-LNC1007 with [^18^F]FDG and [^18^F]AlF-NOTA-FAPI-04 PET/CT in RCC.

**Materials and Methods**: A total of 35 participants, highly suspected to have RCC, were recruited. [^18^F]AlF-LNC1007 and [^18^F]AlF-NOTA-FAPI-04/[^18^F]FDG scans were performed at least one day apart, and both were completed within one week. The Wilcoxon signed-rank test or paired t-test was used to assess differences in tumor uptake and TBR (tumor-to-background ratio) between [^18^F]AlF-LNC1007 and the other two imaging agents. The Spearman correlation coefficient was used to evaluate the correlation between tumor uptake and the expression of FAP and α_v_β_3._

**Results:** The detection rate, sensitivity, and positive predictive value (PPV) of [^18^F]AlF-LNC1007 for RCC primary lesions were significantly higher than those of [^18^F]FDG, at 91% vs. 76%, 100% vs. 85%, and 91% vs. 87%, respectively. Obvious advantages were also seen in metastatic lesions at 94% vs. 34%, 94% vs. 29%, and 100% vs. 100%. Compared to [^18^F]AlF-NOTA-FAPI-04, the corresponding detection rate, sensitivity, and PPV were 98% vs. 90%, 100% vs. 92%, and 98% vs. 98% for primary lesions, and 89% vs. 78%, 89% vs. 93%, and 100% vs. 82% for metastatic lesions. The uptake and TBR of [^18^F]AlF-LNC1007 in both primary and metastatic lesions were significantly higher than those of [^18^F]FDG (all P < 0.001). The uptake of [^18^F]AlF-LNC1007 showed a moderate to high positive correlation with the expression levels of α_v_β_3_ and the combined expression of FAP and α_v_β_3_ (r = 0.756, P = 0.0003; r = 0.678, P = 0.0002) and a low positive correlation with FAP expression alone (r = 0.389, P = 0.014). The uptake of [^18^F]AlF-NOTA-FAPI-04 showed a low to moderate positive correlation with FAP expression and the combined expression of FAP and α_v_β_3_ (r = 0.570, P = 0.0002; r = 0.408, P = 0.010), and no correlation with α_v_β_3_ expression alone (r = 0.262, P = 0.107).

**Conclusion:** [^18^F]AlF-LNC1007 demonstrated significantly higher diagnostic efficacies and uptake in primary and metastatic renal cell carcinoma (RCC) compared to FDG PET/CT. Additionally, [^18^F]AlF-LNC1007 exhibited higher diagnostic efficacies and uptake in primary RCC than [^18^F]AlF-NOTA-FAPI-04 PET/CT. While these findings suggest potential diagnostic advantages, further studies are needed to fully evaluate its diagnostic efficacy compared to the standard of treatment.

## Introduction

RCC (Renal cell carcinoma, RCC) is the most common type of kidney tumor and accounts for about 2-3% of adult malignancies 1. The incidence rate in men is 1.5-1.6 times higher than in women 2. Approximately 30% of patients have multiple site metastases at the time of RCC diagnosis 3, and 20-40% of patients suffer from distant metastasis with very poor prognosis or local recurrence after radical nephrectomy 4. Currently, conventional anatomical imaging cannot examine the entire body in detail and often misses local or distant occult tumors. In contrast, molecular imaging such as PET can provide a large amount of diagnostic information about the entire body, which is particularly important today as individualized treatment is increasingly emphasized.

It is well known that ^18^F-FDG positron emission tomography/X-ray computed tomography (PET/CT) has difficulty distinguishing RCC from benign kidney tumors and may easily miss some lesions 5. Additionally, because glucose transporter 1 is lowly expressed in RCC cells, FDG-PET may not be an effective diagnostic tool for RCC 6.

New targeted fibroblast activation protein (FAP) radiolabeled FAP inhibitor (FAPI) PET/CT imaging has achieved promising results in the diagnosis and treatment of many solid cancers 7. However, research on FAPI PET in kidney tumors is scarce and mostly limited to case reports. The arginine-glycine-aspartate (RGD) peptide targeting integrin α_v_β_3_ and its derivatives PET/CT molecular imaging is also widely used to differentiate malignant lesions and assess tumor angiogenesis and metastasis 8. α_v_β_3_ and its binding ligand RGD are attractive targets for therapy and imaging 9. RGD PET may even be a unique non-invasive tool for evaluating tumor invasiveness and metastatic potential and, thus, may become a new prognostic marker 10.

The application of dual-targeting tracers has shown promising clinical potential compared to single-target tracers, demonstrating advantages in the detection of various tumors 11. [^68^Ga]Ga-FAPI-RGD (denoted as [^68^Ga]Ga-LNC1007) is a new heterodimeric PET tracer targeting both FAP and α_v_β_3_ 12. Compared to their respective monomers, the metabolic stability of peptide multimers is generally improved 13, and [^68^Ga]Ga-LNC1007 has been reported as a promising PET agent 14 used for imaging various cancers 12, 15. Different from the aforementioned studies, we used the radionuclide ^18^F instead of ^68^Ga. Its advantages include a longer half-life, making it more suitable for extended circulation and tumor retention, and higher resolution due to its lower energy compared to ^68^Ga (0.65 MeV vs. 1.90 MeV). The purpose of this study is to compare the application value of [^18^F]AlF-LNC1007, [^18^F]FDG, and [^18^F]AlF-NOTA-FAPI-04 PET/CT in RCC.

## Materials and methods

### Patients

This prospective clinical study was approved by the Ethics Committee of Sichuan Provincial People's Hospital and registered at ClinicalTrials.gov (NCT05976607). Participants highly suspected of having RCC were recruited from September 2023 to March 2024. Inclusion criteria: (1) RCC suspected by routine imaging and clinical examination without a history of kidney disease; (2) PET/CT examinations with different imaging agents performed within one week and confirmed by pathology and immunohistochemistry. Exclusion criteria: (1) pregnancy or lactation; (2) presence of a second primary tumor; (3) severe diseases (such as liver or kidney dysfunction, active tuberculosis, etc.). Written informed consent was obtained from each patient.

**Figure 1:**
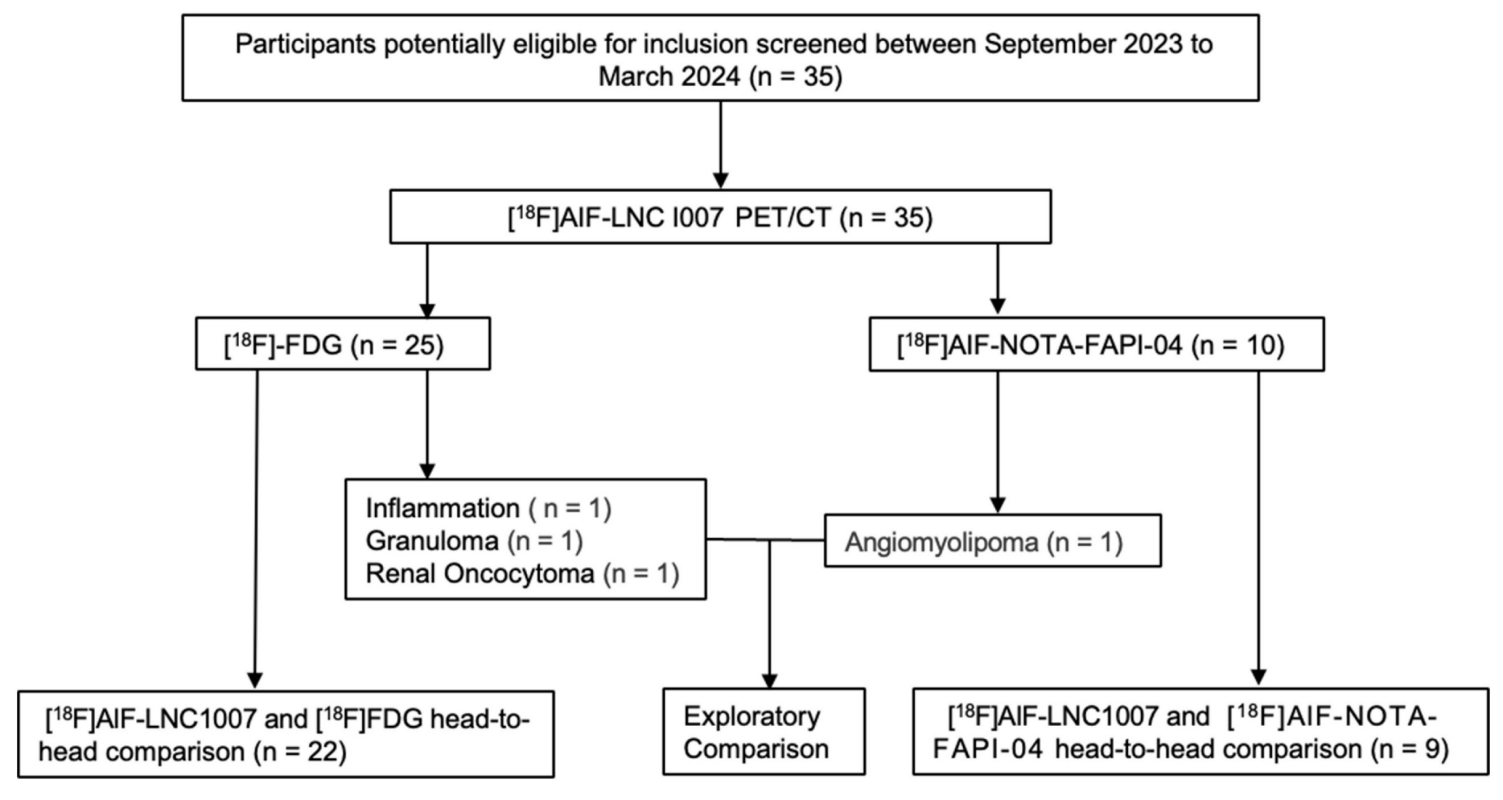
The study workflow diagram.

A total of 35 patients were included, 24 males and 11 females, aged 49-83 years, with a median age of 63 years. All patients were pathologically confirmed, with 31 diagnosed with RCC (29 clear cell carcinomas and 2 chromophobe cell carcinomas). The remaining four cases that were non-RCC served as control observation cases, diagnosed as angiomyolipoma, local inflammation granuloma, and renal oncocytoma, respectively (Figure 1). Among the 31 RCC patients, 12 had distant metastases. 22 underwent additional [^18^F]FDG, and 9 underwent additional [^18^F]AlF-NOTA-FAPI-04 PET/CT for comparison with [^18^F]AlF-LNC1007 (Table 1).

### Radiopharmaceuticals

The FAPI-RGD precursor was provided by Lannacheng Biotechnology Co., Ltd., Yantai, China. [^18^F]AlF-labeled FAPI-RGD ([^18^F]AlF-LNC1007) was synthesized using an Allinone module (Trasis, Ans, Belgium), The [^18^F]AlF- radiolabeling of the heterodimer was performed using [^18^F]fluoride and a 0.5M NaOAc buffer at pH 4, which included AlCl3 and the peptide LNC1007. The overall synthesis duration was around 30-45 min. The radiochemical yield (RCY, decay-corrected) was 50 ± 6.8% (n = 8) for the manual method and 36.6 ± 2.4% (n = 4) for the automated process 16 (More details can be found in the Supplemental Materials). NOTA-FAPI-04 was purchased for the study. Radiolabeling followed standard procedures reported in preclinical studies. Radiochemical purity exceeded 95%, and sterility was confirmed before injection. [^18^F]FDG preparation followed standard clinical practice.

### PET/CT acquisition

A dedicated PET/CT scanner was used, with a tube voltage of 120 KV and tube current using automated mAs for low-dose CT scanning. PET acquisition used Flow technology, and image reconstruction adopted TrueX+TOF, with image registration using specialized software (TrueD software, Siemens). [^18^F]AlF-LNC1007 and [^18^F]AlF-NOTA-FAPI-04/[^18^F]FDG scans were performed at least one day apart, and both were completed within one week. No special preparation was required before the injection of [^18^F]AlF-LNC1007 and [^18^F]AlF-NOTA-FAPI-04. Both tracers were administered according to a dose calculated according to the patient's weight, using 1.8-2.2 MBq [0.05-0.06 mCi]/kg. Patients were scanned 90 min post-injection of [^18^F]AlF-LNC1007 and 60 min post-injection of [^18^F]AlF-NOTA-FAPI-04. For [^18^F]FDG, fasting for at least 6 hours was required before injection, and blood glucose needed to be below 140 mg/dL. [^18^F]FDG scans were performed 60 min after intravenous injection of 5.55 MBq/kg [^18^F]FDG.

### Image analysis

Two nuclear medicine physicians independently evaluated images blinded to clinical data, with consensus defining tumor characterization (benign/malignant). Discrepancies were resolved by a senior radiologist. Positive lesions were defined as focal tracer uptake exceeding background, excluding physiological uptake (thyroid/kidneys/pancreas/salivary glands/bladder) 15, 16. Indeterminate lesions underwent clinical correlation, cross-sectional imaging review, or delayed-phase imaging 15. For large tumors, a PET-guided sampling strategy was used to target areas of high, intermediate, and low metabolic activity in collaboration with surgical teams. This ensured an accurate correlation between ^18^F-FDG uptake and histopathology, improving diagnostic accuracy for personalized treatment. In cases where tumors were larger than 5 cm, we subdivided the pathological specimens to better capture the heterogeneous nature of these tumors and thus effectively increase the overall sample size for analysis. Specifically, each large tumor was divided into multiple independent "lesions" or sections. For tumors showing relatively homogenous tracer uptake, we collected 2-4 samples (each approximately 1 cm³) from different areas representing high, intermediate, and low metabolic activity. For tumors with visibly varied uptake patterns, we performed multipoint sampling, focusing particularly on the hypermetabolic regions. Also, a 1cm diameter sphere was drawn in the upper, middle, and lower parts of the contralateral normal renal parenchyma to obtain SUV_mean_, and the average of the three SUV_mean_ values was taken as the average SUV_mean_. TBR was obtained by SUV_max_/avgSUV_mean_. For metastatic lesions, TBR was calculated using the SUV_mean_ of the diseased side muscle as the background.

### Histological and follow-up

40 RCC primary lesions and 45 metastases lesions underwent routine hematoxylin-eosin (HE) staining of biopsy or surgical specimens. Lesions without pathological results were confirmed by imaging findings (BS, CT, MRI, or PET/CT) and clinical follow-up (≥6 months). Typical malignant characteristics were confirmed by multimodal medical imaging, defined as significant progression observed in follow-up imaging, or a notable reduction in tumor size after treatment. Immunohistochemical (IHC) detection of FAP (α antibody ab207178, Abcam, 1:250) and integrin α_v_β_3_ (ab210515, Abcam, 1:400) expression was performed on paraffin sections of primary tumors after surgery 17. Two experienced pathologists independently interpreted the IHC analyses, blinded to the clinical diagnoses and other imaging results. Any discrepancies were resolved by consensus with a third pathologist (with 20 years of experience). The FAP and integrin α_v_β_3_ expression, scoring staining intensity (0, negative; 1, weakly positive; 2, positive; and 3, strongly positive) and the proportion of positive staining (1, 0% ≤ positive staining ≤ 25%; 2, 25% < positive staining ≤ 50%; 3, 50% < positive staining ≤ 75%; 4, 75% < positive staining ≤ 100%). The IHC score = staining intensity × proportion of positive staining; the total scores for FAP and integrin α_v_β_3_ were calculated based on their respective scores.

### Statistical analysis

Data analysis was performed using IBM SPSS 26.0 software and GraphPad Prism 9 software. Quantitative data were expressed as mean ± standard deviation. The McNemar test was used to compare the detection rates between [^18^F]AlF-LNC1007 and [^18^F]FDG/[^18^F]AlF-NOTA-FAPI-04 PET/CT. The Wilcoxon signed-rank test was used to assess differences in tumor uptake and TBR between [^18^F]AlF-LNC1007 and [^18^F]FDG/[^18^F]AlF-NOTA-FAPI-04 PET/CT. The Spearman correlation coefficient was used to evaluate the correlation between tumor uptake and FAP and integrin α_v_β_3_ expression. P-values < 0.05 were considered statistically significant.

## Results

### Comparison of diagnostic efficacy between [^18^F]AlF-LNC1007 and [^18^F]FDG PET/CT, [^18^F]AlF-LNC1007 and [^18^F]AlF-NOTA-FAPI-04

**Figure 2:**
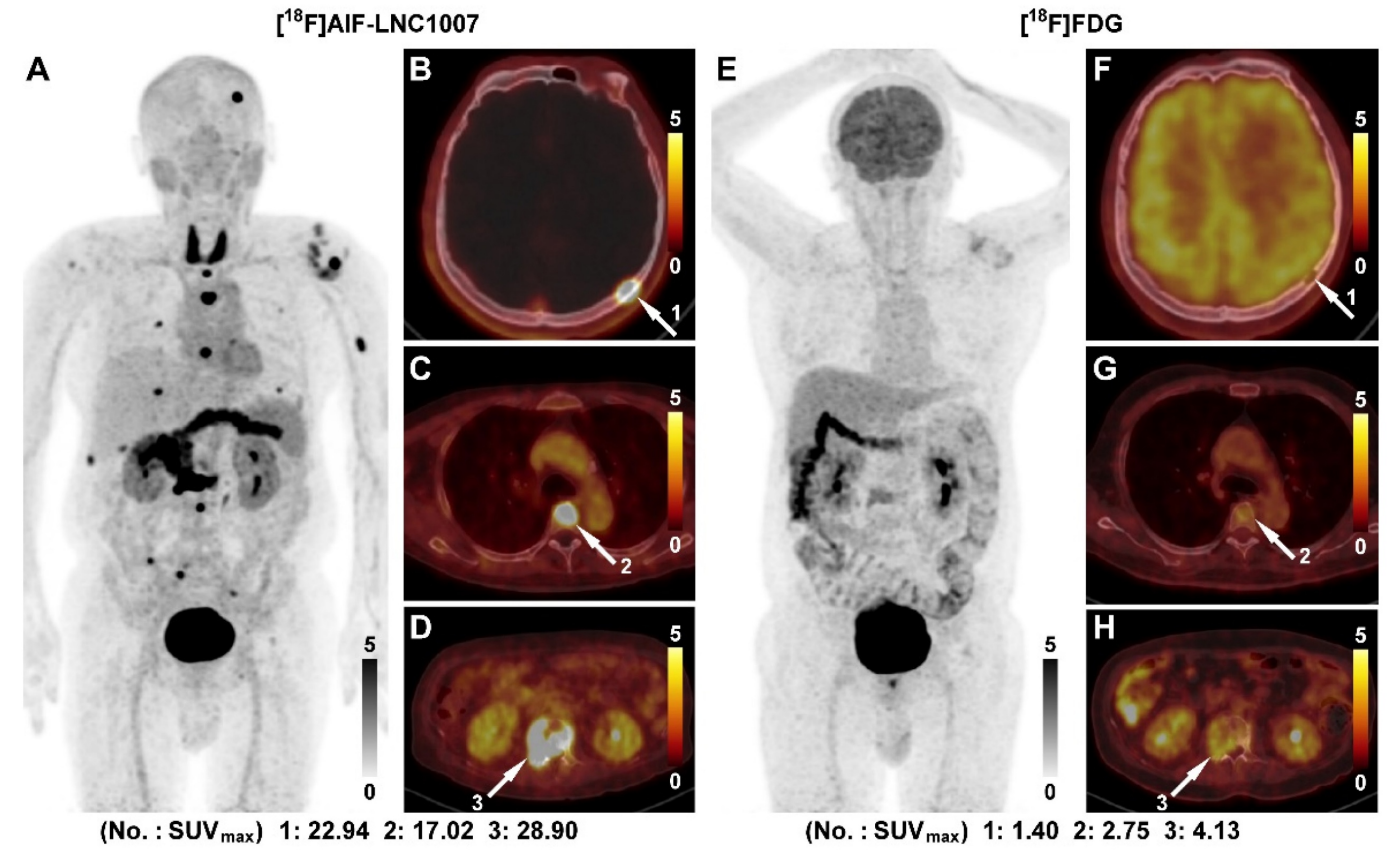
A 56-year-old male was diagnosed with clear cell RCC of the right kidney with multiple bone metastases (A). [^18^F]AlF-LNC1007 PET revealed intense radiotracer uptake in all metastases (B-D, white arrow). [^18^F]FDG PET (E) identified very few lesions and had weak uptake, as seen in bone metastases (F-H, white arrow).

In the comparison of 22 cases between [^18^F]AlF-LNC1007 and [^18^F]FDG, 45 primary lesions were clearly diagnosed (4 discrepant lesions were resolved through consensus). [^18^F]AlF-LNC1007 detected 41 lesions, with 4 false positives, while [^18^F]FDG detected 34 lesions, with 5 false positives and 6 false negatives. Among the 7 patients with confirmed metastasis, there were a total of 70 metastatic lesions (11 discrepant lesions were resolved), including 27 in the thyroid, abdomen, and muscles, 21 bone metastases, 19 lung and pleural metastases, and 3 adrenal gland metastases. Among these metastatic lesions, [^18^F]AlF-LNC1007 detected 66, with 4 false negatives, while [^18^F]FDG detected only 24, with 46 false negatives (Figure 2). The detection rate, sensitivity, and PPV of [^18^F]AlF-LNC1007 for RCC primary lesions were significantly higher than those of [^18^F]FDG, at 91% vs. 76%, 100% vs. 85%, and 91% vs. 87%, respectively (P < 0.05). Obvious advantages were also seen in metastatic lesions, at 94% vs. 34% (P = 0.048), 94% vs. 29% (P = 0.0003), and 100% vs. 100% (Table 2).

**Figure 3:**
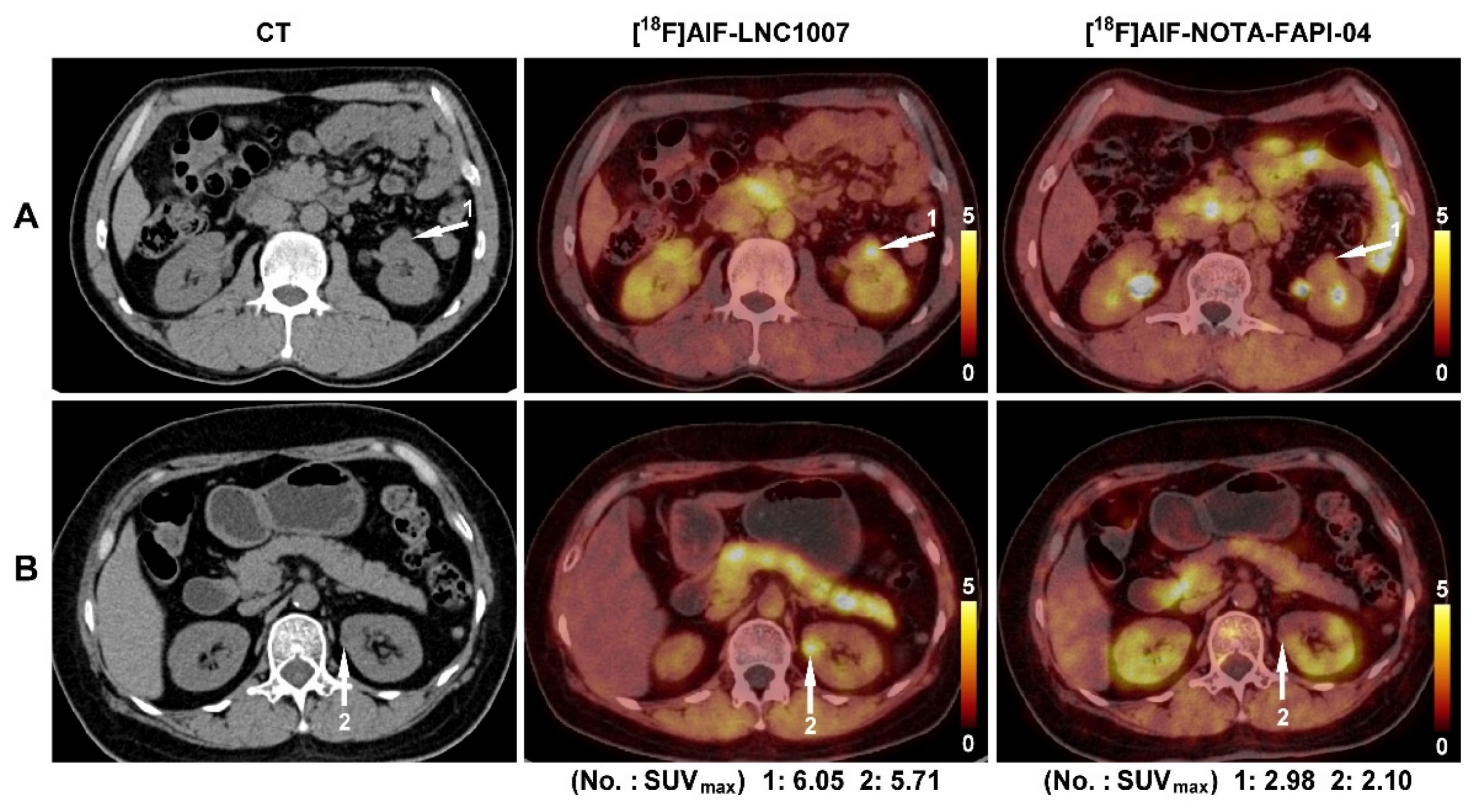
A 58-year-old male(A) and a 62-year-old female (B) were both diagnosed with clear cell RCC of the left kidney. Both lesions had high uptake of [^18^F]AlF-LNC1007 but weak uptake of [^18^F]AlF-NOTA-FAPI-04.

In the comparison of 9 cases (5 patients had larger tumors) between [^18^F]AlF-LNC1007 and [^18^F]AlF-NOTA-FAPI-04, there were 63 clearly diagnosed primary lesions (5 discrepant lesions were resolved through consensus). [^18^F]AlF-LNC1007 detected 62 lesions, with 1 false positive, while [^18^F]AlF-NOTA-FAPI-04 detected 57 lesions, with 1 false positive and 5 false negatives (Figure 3). Among the 5 patients with confirmed metastasis, there were a total of 18 metastatic lesions (2 discrepant lesions were resolved), including 8 bone metastases, 8 lymph node metastases, 1 breast metastasis, and 1 muscle metastasis. Among these metastatic lesions, [^18^F]AlF-LNC1007 detected 16, with 2 false negatives, while [^18^F]AlF-NOTA-FAPI-04 detected only 14, with 3 false positives and 1 false negative. The detection rate, sensitivity, and PPV of [^18^F]AlF-LNC1007 for RCC primary lesions were significantly higher than those of [^18^F]AlF-NOTA-FAPI-04, at 98% vs. 90% (P = 0.052), 100% vs. 92% (P = 0.022), and 98% vs. 98%, respectively. For metastatic lesions, the corresponding percentages were 89% vs. 78% (P = 0.371), 89% vs. 93% (P = 0.658), and 100% vs. 82% (P = 0.078) (Table 2).

### Comparisons of lesion uptake between [^18^F]AlF-LNC1007 and [^18^F]FDG PET/CT, and [^18^F]AlF-LNC1007 and [^18^F]AlF-NOTA-FAPI-04

In all primary and metastatic lesions in the 22 patients compared between [^18^F]AlF-LNC1007 and [^18^F]FDG, [^18^F]AlF-LNC1007 showed higher SUV_max_, SUV_mean_, and TBR than [^18^F]FDG, with values of 8.31 ± 3.56 vs. 4.82 ± 3.54, 4.26 ± 1.39 vs. 2.80 ± 1.10, and 2.68 ± 1.15 vs. 1.34 ± 0.65, respectively (all P < 0.001) (Table 3).

**Figure 4:**
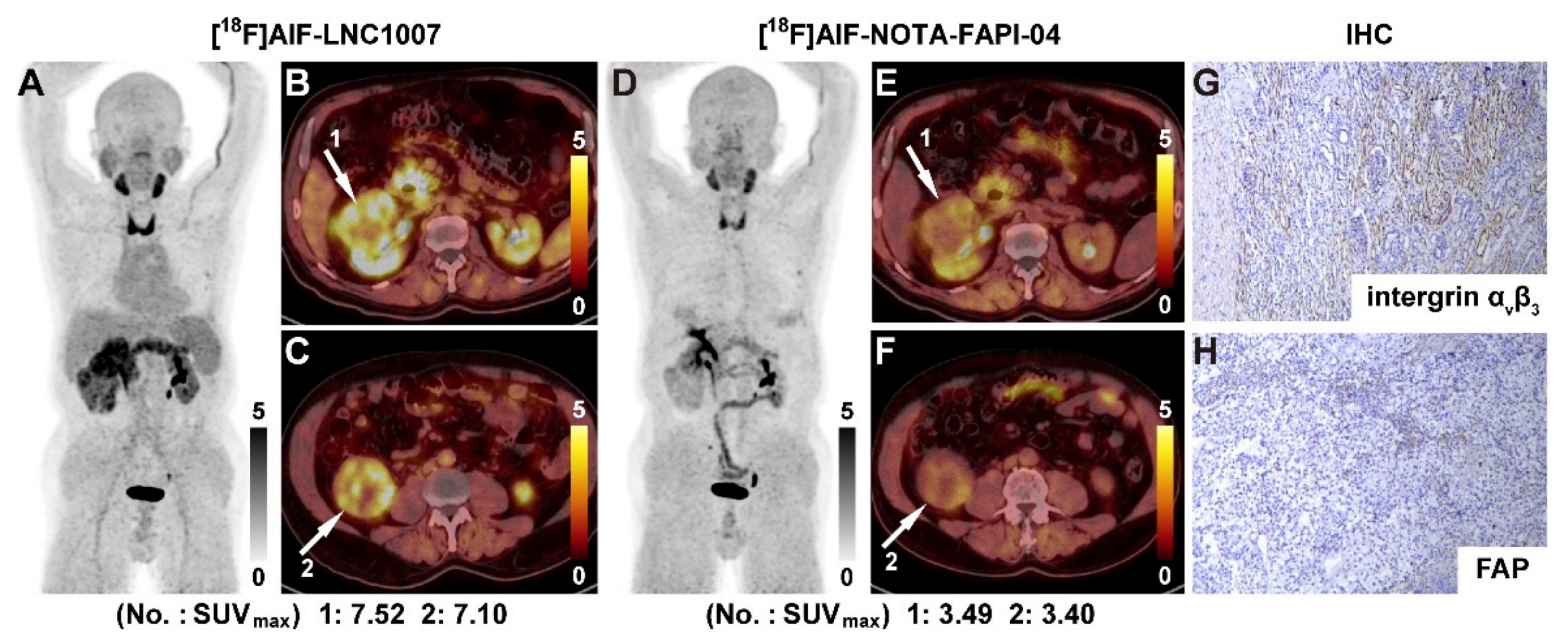
A 69-year-old male was diagnosed with clear cell RCC. [^18^F]AlF-LNC1007 PET(A) and PET/CT (B-C, white arrow) depicted intense tracer uptake in the primary tumor. [^18^F]AlF-NOTA-FAPI-04 images showed weak uptake in corresponding lesions (D-F). The immunohistochemical staining revealed strong integrin α_v_β_3_ (G) and weak FAP expression (H). The IHC scores were 3 and 1, respectively.

In the primary lesions of the 9 patients compared between [^18^F]AlF-LNC1007 and [^18^F]AlF-NOTA-FAPI-04, [^18^F]AlF-LNC1007 had higher SUV_max_ and SUV_mean_ than [^18^F]AlF-NOTA-FAPI-04 (Figure 4), with values of 7.02 ± 2.59 vs. 4.50 ± 2.02, 5.82 ± 2.59 vs. 3.76 ± 2.51 (all P < 0.001). The TBR was lower than [^18^F]AlF-NOTA-FAPI-04 (2.03 ± 0.88 vs. 2.15 ± 0.95), but the difference was not statistically significant (P = 0.091). In metastatic lesions, [^18^F]AlF-LNC1007 had lower SUV_max_ and SUV_mean_ than [^18^F]AlF-NOTA-FAPI-04, with values of 3.25 ± 0.96 vs. 4.04 ± 1.67, 2.06 ± 0.52 vs. 2.58 ± 0.93 (all P < 0.05), while the TBR was higher than [^18^F]AlF-NOTA-FAPI-04 (2.57 ± 0.81 vs. 1.98 ± 1.16, P = 0.005) (Table 3).

To compare the differences between [^18^F]FDG and [^18^F]AlF-NOTA-FAPI-04, the SUV_max_, SUV_mean_, and TBR of the two groups were tested as independent samples using the Wilcoxon test. The results showed no statistically significant differences between the two groups, with P-values of 0.230, 0.844, and 0.072, respectively (Table 3).

In the four non-RCC patients, the angiomyolipoma patient underwent both [^18^F]AlF-LNC1007 and [^18^F]AlF-NOTA-FAPI-04 PET/CT, with SUV_max_ of 5.65 and 5.87, respectively. The other three patients underwent both [^18^F]AlF-LNC1007 and ^18^F-FDG, showing SUV_max_ of 14.68 and 6.74 in inflammation, 5.25 and 2.61 for eosinophilic cell tumor, and 2.38 and 3.57 for granuloma, respectively (Table S1).

### Correlation between the uptake of [^18^F]AlF-LNC1007/[^18^F]AlF-NOTA-FAPI-04 and α_v_β_3_ expression, FAP expression, and α_v_β_3_ + FAP expression

**Figure 5:**
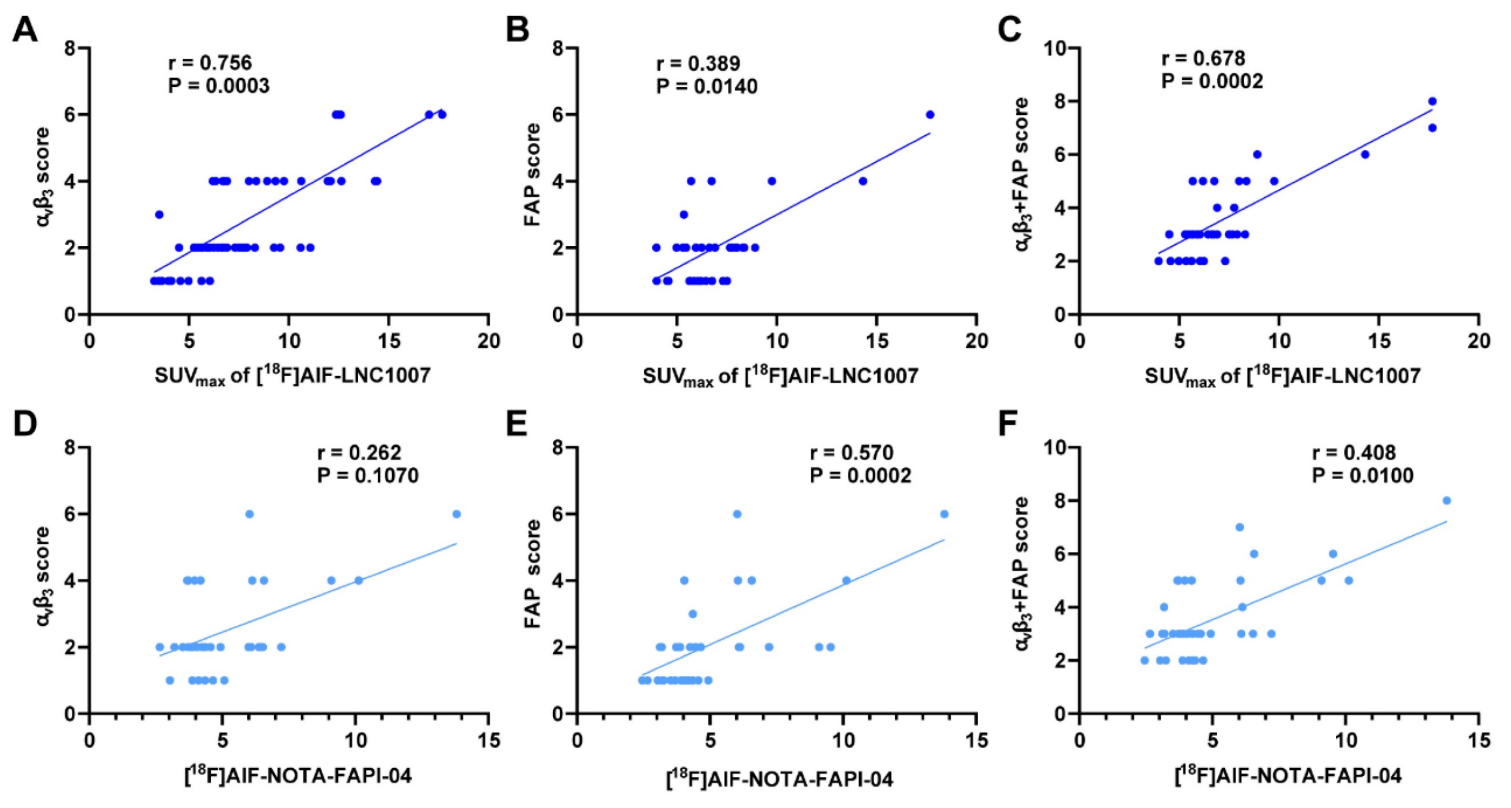
[^18^F]AlF-LNC1007 SUV_max_ were moderately to highly correlated with integrin α_v_β_3_ and integrin α_v_β_3_ + FAP expression (A, C) but weakly correlated with FAP expression alone (B). [^18^F]AlF-NOTA-FAPI-04 SUV_max_ was not correlated with integrin α_v_β_3_ (D) but was weakly and moderately correlated with FAP and FAP + integrin α_v_β_3_ expression (E-F).

**Figure 6:**
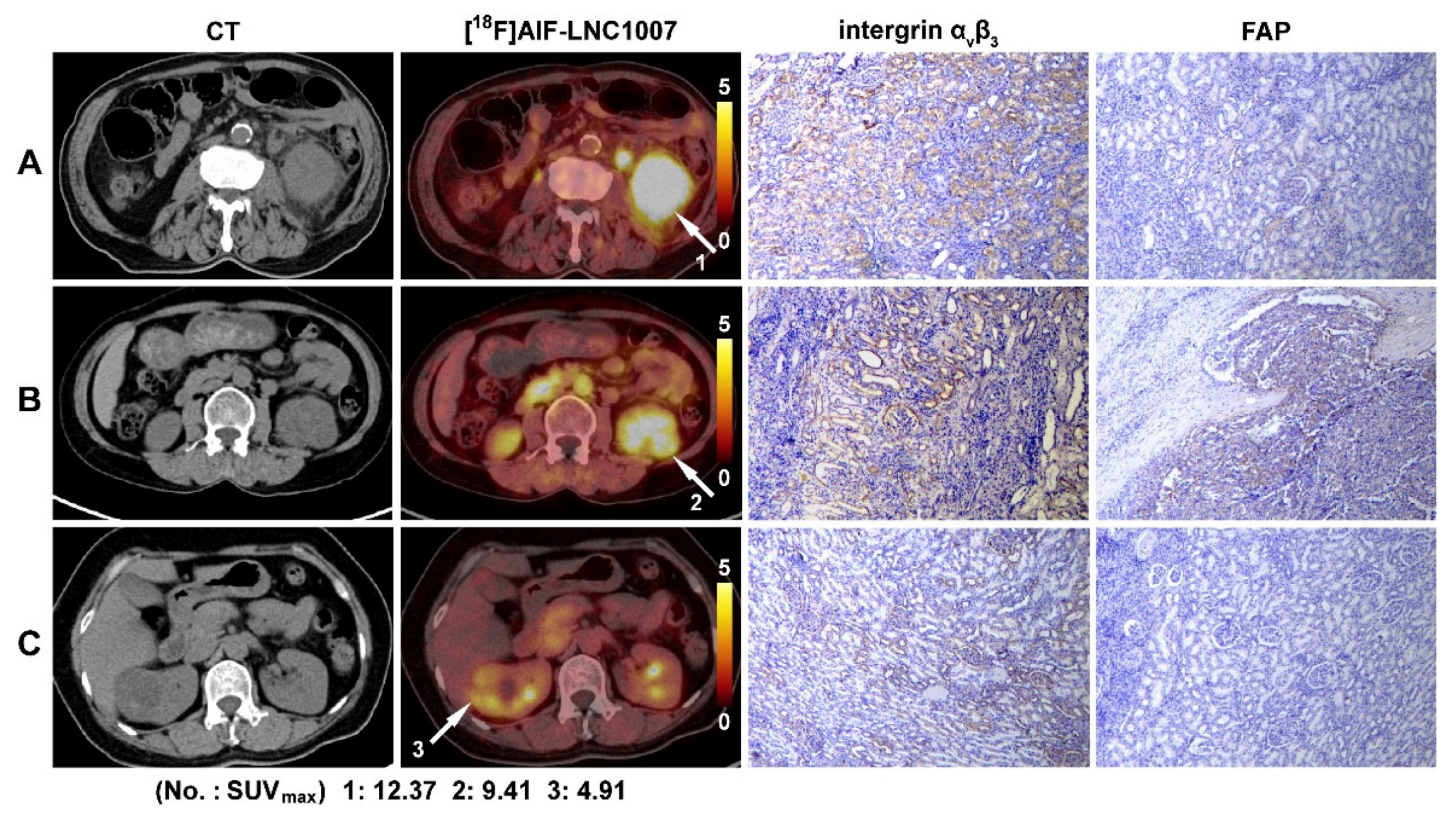
[^18^F]AlF-LNC1007 PET/CT images and immunohistochemical staining in three patients with clear cell RCC. Primary tumor sites are indicated by the white arrow, and the SUV_max_ was 8.7 (A), 5.7 (B), and 6.2 (C). The corresponding IHC scores of the integrin α_v_β_3_ expression were 6, 4, and 2, and the FAP scores were 1, 2 and 0, respectively.

To increase the sample size, pathological sections of larger tumors were decomposed. A total of 64 sections from 31 patients were analyzed (7 discrepant sections were resolved). Integrin α_v_β_3_ staining showed 6 sections (9.4%) with no expression, 15 sections (23.4%) with weak expression, 23 sections (35.9%) with moderate expression, and 20 sections (31.3%) with strong expression. The proportions of positive staining scores of 1-4 were 17.6%, 30.4%, 28.9%, and 23.1%, respectively. The IHC score range was 0-9. The SUV_max_ of [^18^F]AlF-LNC1007 showed a moderate to high positive correlation with the integrin α_v_β_3_ IHC score and the combined FAP and integrin α_v_β_3_ IHC scores (r = 0.756, P = 0.0003; r = 0.678, P = 0.0002) (Figure 5A, C) (Figure 6). However, it showed a low positive correlation with the FAP IHC score (r = 0.389, P = 0.014) (Figure 5B).

Among the 9 patients who underwent both [^18^F]AlF-LNC1007 and [^18^F]AlF-NOTA-FAPI-04 PET/CT, 39 tissue sections were obtained for pathology (4 discrepant sections were resolved). FAP staining showed 7 sections (17.9%) with no expression, 17 sections (43.6%) with weak expression, 11 sections (28.2%) with moderate expression, and 4 sections (10.3%) with strong expression. The proportions of positive staining scores of 1-4 were 30.7%, 32.8%, 19.9%, and 16.6%, respectively. The IHC score range was 0-8. The SUV_max_ of [^18^F]AlF-NOTA-FAPI-04 showed no correlation with the integrin α_v_β_3_ IHC score (r = 0.262, P = 0.107) (Figure 5D). However, it demonstrated a low to moderate positive correlation with the FAP IHC score and the combined FAP and integrin α_v_β_3_ IHC scores (r = 0.570, P = 0.0002; r = 0.408, P = 0.010) (Figure 5E-F).

## Discussion

RCC is a highly vascular tumor with unpredictable progression, and its incidence and mortality rates have been increasing worldwide at a rate of about 2-3% per decade 18. Early diagnosis and precise staging are crucial for the prognosis of RCC. However, conventional imaging examinations, including FDG PET, have shown poor performance in RCC diagnosis 19. ^18^F-FDG PET can detect only 40% of local and metastatic disease in RCC patients 20, which is consistent with the results in this study, where FDG was found to have numerous false-negative lesions. FDG PET's SUV_max_, SUV_mean_, and TBR were all found to be significantly lower than those of [^18^F]AlF-LNC1007 (all P < 0.001), aligning with recent reports on [^68^Ga]Ga-LNC1007 in RCC 17. This could be due to the low glucose metabolism potential of RCC cells 6 and the physiological excretion of [^18^F]FDG from the kidneys, which may obscure kidney lesions 21.

Cancer-associated fibroblasts (CAFs) can comprise up to 90% of the stromal tissue in certain tumors 22. However, systematic studies on FAPI in RCC are rarely reported 23. In this study, the [^18^F]AlF-NOTA-FAPI-04 group had lower accuracy in diagnosing primary and metastatic RCC lesions compared to [^18^F]AlF-LNC1007, with relatively more false-negative and false-positive lesions. The uptake of [^18^F]AlF-NOTA-FAPI-04 in primary lesions was significantly lower than in the [^18^F]AlF-LNC1007 group, consistent with literature reports that RCC cases show lower FAPI uptake due to low FAP expression 24. Masatoshi Hotta et al. confirmed that clear cell RCC, the most common type of RCC, typically exhibits mild FAPI uptake 23 and a weak fibroblast response, making RCC one of the tumors with the lowest ^68^Ga-FAPI uptake 19.

Integrin α_v_β_3_ is highly expressed in activated endothelial cells during tumor angiogenesis 25. RGD PET can non-invasively and selectively target α_v_β_3_ integrin 26, and the SUV of RGD PET is significantly correlated with α_v_β_3_ expression 27, 28. [^18^F]fluciclatide (formerly known as [^18^F]AH111585) has shown good imaging and tolerability characteristics, allowing for high TBR imaging in RCC, with uptake moderately to strongly correlated with integrin expression in IHC 9. Other studies have indicated that [^18^F]FPPRGD2 PET/CT has lesion detectability in mRCC patients 29 and can reflect tumor-associated integrin α_v_β_3_ activity in RCC 30.

Our results demonstrate that [^18^F]AlF-LNC1007 exhibits high diagnostic efficacy and uptake in primary RCC lesions, consistent with previous studies 31, 32. However, its uptake in metastatic lesions was marginally lower compared to [^18^F]AlF-NOTA-FAPI-04. Potential explanations include the limited number of metastatic lesions and inherent tumor heterogeneity, which may introduce sampling bias and limit generalizability to all metastatic scenarios. Additionally, lesion characteristics such as size and progression stage might influence uptake patterns; in our cohort, all bone metastases were small lesions. Prior studies have identified cancer-associated fibroblasts (CAFs) in bone metastases as small as 1-2 mm 33, and Kömek et al. reported robust detection of bone metastases using [^68^Ga]Ga-DOTA-FAPI-04 34. Notably, approximately 30% of early metastatic lesions lack overt osteolytic or osteoblastic changes 35, while integrin α_v_β_3_ overexpression is predominantly observed in mature osteoclasts 36. These observations align with findings suggesting that α_v_β_3_ expression levels in bone metastases may correlate with lesion maturity and angiogenesis. For instance, studies indicate progressive increases in angiogenic factor expression during advanced cancer stages 37. Further investigations are required to clarify the temporal dynamics of α_v_β_3_ expression during metastatic progression.

Immunohistochemical analysis revealed that integrin α_v_β_3_ was predominantly expressed at moderate to strong levels in RCC tissues, while FAP exhibited weak expression. These findings align with recent reports 17, which indicate that most RCCs demonstrate moderate-to-strong α_v_β_3_ expression but limited FAP positivity, with some lesions being entirely FAP-negative. The [^18^F]AlF-NOTAFAPI-04 results showed a moderate correlation with the IHC scoring, which is related to tumor heterogeneity, the small sample size, and the location of the samples. The observed correlation between [^18^F]AlF-LNC1007 SUV_max_ and α_v_β_3_ expression, as well as combined α_v_β_3_+FAP expression, suggests a potential association between tracer uptake and angiogenic activity. The stronger correlation between tracer uptake and α_v_β_3_ expression (r = 0.756) compared to the combined FAP and α_v_β_3_ expression (r = 0.678) suggests that α_v_β_3_ may be the dominant driver of uptake in RCC. This has important implications for the clinical application of LNC1007. In the context of RCC, particularly ccRCC which is highly angiogenic, α_v_β_3_ integrin is known to be overexpressed, and its expression is associated with tumor invasiveness and metastatic potential.

This opens the possibility for LNC1007 to support patient stratification by identifying tumors with high α_v_β_3_ expression, which may be more responsive to anti-angiogenic targeted therapies. Furthermore, the tracer could potentially aid in therapy monitoring, providing early insight into treatment efficacy by tracking changes in integrin expression before anatomical changes become apparent on CT or MRI. Given that RCC recurrence can be driven by angiogenic escape mechanisms, LNC1007 may also prove valuable for early detection of progression or recurrence, even in lesions that remain morphologically stable.

While we did not include a direct comparison with an RGD-based tracer in this study, our findings suggest that [¹⁸F]AlF-LNC1007 exhibits a similar uptake pattern, driven primarily by α_v_β_3_ interaction, and is therefore likely noninferior in reflecting relevant aspects of RCC biology. That said, the contribution of FAP targeting cannot be excluded, and this dual-targeting capability may offer added value in future applications, such as treatment monitoring or in RCC subtypes with a more prominent stromal component.

Additionally, our data corroborate previous studies 12, 15 demonstrating that the heterodimeric tracer LNC1007 exhibits enhanced tumor accumulation and prolonged retention compared to its monomeric counterparts. These pharmacokinetic properties highlight its diagnostic advantages, particularly in lesion detection and staging. While preclinical evidence supports the potential of LNC1007 as a theranostics agent when labeled with therapeutic radionuclides (e.g., ^177^Lu/^90^Y/^225^Ac), its therapeutic efficacy and safety profile in RCC remain to be systematically evaluated in dedicated clinical studies.

Our study has several limitations. The number of cases in this cohort study is relatively small, especially in the comparison group of [^18^F]AlF-LNC1007 and [^18^F]AlF-NOTA-FAPI-04, which comprised only 18 metastatic lesions and, therefore, may lead to data bias. This cohort mainly consisted of clear cell RCC (29/31), and we did not include other pathological subtypes of RCC for subgroup analysis. The use of immunohistochemistry allowed only for semi-quantitative analysis of FAP and α_v_β_3_ expression. Single immunohistochemical slides and PET images do not completely overlap spatially, so the immunohistochemical slides may not have represented the regions with the highest uptake of imaging agents.

## Conclusion

In this prospective study, [^18^F]AlF-LNC1007 has significantly higher tumor detection rates and uptake in primary and metastatic ccRCC patients compared to FDG PET/CT. [^18^F]AlF-LNC1007 also showed higher tumor detection rates and uptake in primary ccRCC patients compared to [^18^F]AlF-NOTA-FAPI-04 PET/CT. It holds promise for diagnosing RCC, evaluating the efficacy of therapy, and highlighting its potential as a targeted therapeutic carrier for treating advanced RCC patients.

## Supplementary Material

Supplementary materials and methods, table.

## Figures and Tables

**Table 1:** Summary of Patient Characteristics

Characteristic	Value
No. of patients	35
Median Age(y)	63(49-83)
Gender	
Male	23
Female	12
Diagnosis	
Clear cell carcinoma	29
Chromophobe cell tumor	2
Renal oncocytoma	1
AML	1
Granuloma	1
Inflammation	1
PET/CT	
LNC1007 *vs.* FDG	22
LNC1007 *vs.* FAPI-04	9

AML=angiomyolipoma, FAPI=fibroblast-activation protein inhibitor, RGD=arginine-glycine-aspartate, FDG=fluorodeoxyglucose, LNC1007= FAPI-RGD (arginine-glycine-aspartate).

**Table 2:** Comarison of diagnostic efficacy from [^18^F]AlF-LNC1007 and [^18^F]FDG PET/CT, [^18^F]AlF-LNC1007 and [^18^F]AlF-NOTA-FAPI-04

		Primary tumors	All metastases
LNC1007 vs.FDG			
	True lesions	45	70
	LNC1007	41	66
	FDG	34	24
	Accuracy (%)	91 vs. 76^*^	94 vs. 34 ^**^
	Sen (%)	100 *vs.* 85^*^	94 *vs.* 29 ^**^
	PPV (%)	91 *vs.* 87^*^	100 *vs.*100
LNC1007 vs.FAPI-04			
	True lesions	63	18
	LNC1007	62	16
	FAPI-04	57	14
	Accuracy (%)	98 vs. 90	89 vs. 78
	Sen (%)	100 *vs*. 92^*^	89 vs. 93
	PPV (%)	98 *vs.* 98	100 *vs.*82

^*^ P < 0.05, ^**^ P < 0.01

**Table 3:** Comparison of SUV_max_, SUV_mean_ and TBR of [^18^F]AlF-LNC1007 and [^18^F]FDG PET/CT, [^18^F]AlF-LNC1007 and [^18^F]AlF-NOTA-FAPI-04

	Group	Primary tumors	All metastases
SUV_max_	LNC1007	8.31 ± 3.56	8.95 ± 4.29
	FDG	4.82 ± 3.54^ a^	2.89 ± 2.65
	*p* value	0.0003	0.0002
SUV_mean_	LNC1007	4.26 ± 1.39	5.61 ± 2.46
	FDG	2.80 ± 1.10^ b^	2.00 ± 1.74
	*p* value	0.0002	0.0004
TBR	LNC1007	2.68 ± 1.15	5.18 ± 4.52
	FDG	1.34 ± 0.65^ c^	2.66 ± 2.20
	*p* value	0.0001	0.0002
SUV_max_	LNC1007	7.02 ± 2.59	3.25 ± 0.96
	FAPI-04	4.50 ± 2.02^ a^	4.04 ± 1.67
	*p* value	0.0003	0.008
SUV_mean_	LNC1007	5.82 ± 2.59	2.06 ± 0.52
	FAPI-04	3.76 ± 2.51^ b^	2.58 ± 0.93
	*p* value	0.0001	0.028
TBR	LNC1007	2.03 ± 0.88	2.57 ± 0.81
	FAPI-04	2.15 ± 0.95^ c^	1.98 ± 1.16
	*p* value	0.091	0.005

^a, b,^ and ^c^ represent the comparison of SUV_max_, SUV_mean_, and TBR between FAPI-04 and FDG using the Wilcoxon test, with P-values of 0.230, 0.844, and 0.072, respectively.

## Abbreviations

RCC: renal cell carcinoma
ccRCC: clear cell renal cell carcinoma
FAP: fibroblast activation protein
PPV: positive predictive value
PET/CT: positron emission tomography/computed tomography
FAPI: FAP inhibitor
RGD: arginine-glycine-aspartate
IHC: Immunohistochemical
CAFs: cancer-associated fibroblasts
HE: hematoxylin-eosin
SUV: standardized uptake value
TBR: tumor-to-background ratio
