# Supplementary material for: Comparative study of [18F]AlF-NOTA-FAPI-RGD and [18F]FDG/[18F]AlF-NOTA-FAPI-04 PET/CT in renal cell carcinoma
Source: Theranostics. 2025 Apr 21;15(12):5790–800. doi: 10.7150/thno.113070 (PMC12068302; doi:10.7150/thno.113070)
Supplement: Supplementary file 1 — Supplementary materials and methods, table. [file thnov15p5790s1.pdf]

**Supplemental Materials**

# Comparative Study of [$^{18}$F]AlF-NOTA-FAPI-RGD and [$^{18}$F]FDG/[$^{18}$F]AlF-NOTA-FAPI-04 PET/CT in Renal Cell Carcinoma

Haiyan Gao[1#], Zhiwei Ma[2#], Ziyang Zhu[1#], Zhichuan Yang[3], Bo Chen[1], Xiaoming Wu[4], Vivianne Jakobsson[5,6,7,8], Yujiao Deng[1], Hao Wang[1], Wei Zhang[1*], Jingjing Zhang[5,6,7,8*]

1. Department of Nuclear Medicine, Sichuan Provincial People's Hospital, School of Medicine, University of Electronic Science and Technology of China, Chengdu 610072, China

2. Department of Urology, Sichuan Provincial People's Hospital, University of Electronic Science and Technology of China, Chengdu, 610072, China

3. Department of Emergency Surgery, The Affiliated Chengdu 363 Hospital of Southwest Medical University, Chengdu 610041, China

4. College of Nuclear Science and Technology, Harbin Engineering University, Harbin 150001, China

5.Department of Diagnostic Radiology, Yong Loo Lin School of Medicine, National University of Singapore, Singapore, 119074, Singapore

6. Theranostics Center of Excellence, Yong Loo Lin School of Medicine, National University of Singapore, 11 Biopolis Way, Helios, Singapore 138667, Singapore

7. Clinical Imaging Research Centre, Centre for Translational Medicine, Yong Loo Lin School of Medicine, National University of Singapore, Singapore 117599, Singapore

8. Nanomedicine Translational Research Program, Yong Loo Lin School of Medicine, National University of Singapore, Singapore, 117597, Singapore

[#]Contributed equally to this work.

**First author:** Haiyan Gao, No.32 West Second Section First Ring Road, Chengdu, Sichuan Province, 610072, China. Tel: +8615934143861. Email: haiyangao330@sina.com.

**For correspondence or reprints, contact** Jingjing Zhang, Department of Diagnostic Radiology, Yong Loo Lin School of Medicine, National University of Singapore, Singapore, 119074, Singapore. Phone +6584353534. E-mail: j.zhang@nus.edu.sg; Wei Zhang, No.32 West Second Section First Ring Road, Chengdu, Sichuan Province, 610072, China. Phone +8618116580701. Email: zhangwscd@uestc.edu.cn.

## Additional Materials and Methods

### The process of radiosynthesis for [$^{18}$F]AlF-LNC1007

The automated synthesis of [$^{18}$F]AlF-LN1007 was performed using an AllInOne synthesis module (Trasis, Ans, Belgium). Initially, [$^{18}$F]fluoride was generated and subsequently purified through a Sep-Pak light QMA cartridge (Waters). The retained [$^{18}$F]fluoride was then eluted using 400 μL saline into the reaction vessel. A reaction mixture was prepared by combining LNC1007 (200 μg dissolved in 700 μL DMSO), aluminum chloride solution (20 μL of 0.2 mM in 0.5 M sodium acetate buffer, pH 4.0), and sodium acetate solution (300 μL of 0.5 M, pH 4.0). This mixture underwent thermal treatment at 100°C for 20 minutes followed by controlled cooling to 40°C.

For purification, the cooled mixture was diluted with deionized water and loaded onto an HLB cartridge (30 mg, Waters). The cartridge underwent three washing cycles with deionized water to eliminate unbound [$^{18}$F]fluoride. The purified product was collected by elution with 2 mL of 50% ethanol, further diluted with 10 mL saline, and filtered through a 0.22 μm sterile membrane into a sterile container. Quality control analysis was conducted via radio-HPLC, using a mobile phase system containing 0.1% trifluoroacetic acid (TFA) in acetonitrile (solvent A) and 0.1% TFA in water (solvent B). A gradient elution profile was applied: 10–60% solvent A over 15 minutes.

**Supplementary Table 1. Information of the 4 non-RCC patients**

| Patient | | | Pathological diagnosis | FAPI-RGD | | FDG | | FAPI | |
|---|---|---|---|---|---|---|---|---|---|
| No. | Gender | Age | | $SUV_{max}$ | TBR | $SUV_{max}$ | TBR | $SUV_{max}$ | TBR |
| 1 | F | 60 | Angiomyolipoma | 5.65 | 1.59 | / | / | 5.87 | 4.48 |
| 2 | F | 59 | Inflammation | 14.68 | 6.74 | 10.87 | 4.01 | / | / |
| 3 | F | 50 | Renal Oncocytoma | 5.25 | 1.45 | 2.61 | 1.10 | / | / |
| 4 | M | 63 | Granuloma | 2.38 | 0.78 | 3.57 | 1.94 | / | / |
